# Frailty index and its association with the onset of postoperative delirium in older adults undergoing elective surgery

**DOI:** 10.1186/s12877-022-03663-7

**Published:** 2023-02-11

**Authors:** Janina Steenblock, Ulrike Braisch, Simone Brefka, Christine Thomas, Gerhard W. Eschweiler, Michael Rapp, Brigitte Metz, Christoph Maurer, Christine A. F. von Arnim, Matthias. L. Herrmann, Sören Wagner, Michael Denkinger, Dhayana Dallmeier

**Affiliations:** 1 Agaplesion Bethesda Clinic, Ulm, Germany; 2 Geriatric Center Ulm/Alb-Donau, Ulm, Germany; 3 Institute for Geriatric Research, University Clinic Ulm, Ulm, Germany; 4 Medical Faculty, Ulm University, Ulm, Germany; 5 Institute of Epidemiology and Medical Biometry, Ulm University, Ulm, Germany; 6 Department of Old Age Psychiatry and Psychotherapy, Klinikum Stuttgart, Stuttgart, Germany; 7 Department of Neurology and Neurophysiology, Medical Center-University of Freiburg, Freiburg, Germany; 8 Department of Anesthesia, Critical Care and Pain Medicine, Beth Israel Deaconess Medical Center, Harvard Medical School, Boston, MA 02215 USA; 9 Geriatric Center at the University Hospital Tuebingen, Tuebingen, Germany; 10 Department of Social and Preventive Medicine, University of Potsdam, Am Neuen Palais 10, 14469 Potsdam, Germany; 11 Geriatric Center Karlsruhe, ViDia Christian Clinics Karlsruhe, Karlsruhe, Germany; 12 Center for Geriatric Medicine and Gerontology, University of Freiburg, Freiburg im Breisgau, Germany; 13 Department of Geriatrics, University Medical Center Göttingen, Göttingen, Germany; 14 Department of Anaesthesiology, Klinikum Stuttgart, Kriegsbergstrasse 60, 70174 Stuttgart, Germany; 15 Department of Epidemiology, Boston University School of Public Health, Boston, USA

**Keywords:** Frailty, Postoperative delirium, Elective surgery

## Abstract

**Background:**

The association of frailty based on the accumulation of deficits with postoperative delirium (POD) has been poorly examined. We aimed to analyze this association in older patients undergoing elective surgery.

**Methods:**

Preoperative data was used to build a 30-item frailty index (FI) for participants of the PAWEL-study. Delirium was defined by a combination of I-CAM and chart review. Using logistic regressions models we analysed the association between frailty and POD adjusting for age, sex, smoking, alcohol consumption, education and type of surgery.

**Results:**

Among 701 participants (mean age 77.1, 52.4% male) median FI was 0.27 (Q1 0.20| Q3 0.34), with 528 (75.3%) frail participants (FI ≥ 0.2). Higher median FI were seen in orthopedic than cardiac surgery patients (0.28 versus 0.23), and in women (0.28 versus 0.25 in men). Frail participants showed a higher POD incidence proportion (25.4% versus 17.9% in non-frail). An increased odds for POD was observed in frail versus non-frail participants (OR 2.14 [95% CI 1.33, 3.44], c-statistic 0.71). A 0.1 increment of FI was associated with OR 1.57 [95% CI 1.30, 1.90] (c-statistic 0.72) for POD. No interaction with sex or type of surgery was detected. Adding timed-up-and-go-test and handgrip strength to the FI did not improve discrimination.

**Conclusion:**

Our data showed a significant association between frailty defined through a 30-item FI and POD among older adults undergoing elective surgery. Adding functional measures to the FI did not improve discrimination. Hence, our preoperative 30-item FI can help to identify patients with increased odds for POD.

**Trial registration:**

PAWEL and PAWEL-R (sub-) study were registered on the German Clinical Trials Register (number DRKS00013311 and DRKS00012797).

**Supplementary Information:**

The online version contains supplementary material available at 10.1186/s12877-022-03663-7.

## Introduction

Delirium is an acute and fluctuating disorder characterized by impaired alertness and reduced consciousness, also associated with worsening of cognitive functions such as attention, memory, orientation, language and awareness [1]. In hospital delirium incidence rates range from 10–82%, with highest rates on ICU and postoperative wards [2]. Incident delirium is a common postoperative complication that occurs in up to 46% of cardiac [2], and up to 24% of orthopedic visceral surgical patients [3]. Delirium has been associated with different adverse clinical outcomes, e.g. postoperative cognitive decline [4], extended length of stay (LOS), institutionalization and a higher mortality rate [5].

Risk factors for delirium are multidimensional and can be classified into predisposing non-modifiable ones like age, stroke or preexisting dementia, and modifiable ones such as drug side effects or environmental factors [2, 6, 7]. Thus, it is expected that by addressing modifiable risk factors clinical practitioners especially in surgical departments would be able to optimize the clinical management for those patients identified with high risk of developing postoperative delirium (POD). Frailty is defined as a clinical syndrome with increased vulnerability towards an external stressor leading to increased risk of adverse outcomes [8]. Although available systematic reviews have identified frailty as a predisposing factor for delirium, limitations related to the study sample size and the heterogeneity of the frailty explanatory models have been mentioned [9–11]. The so-called model of deficit accumulation allows the calculation of a frailty index (FI), which represents the individual proportion of potential, multidimensional deficits [12].

So far, the association between frailty and delirium has been evaluated mostly in patients admitted to general medicine wards or in mixed populations [13, 14]. Literature on patients admitted only to surgical departments is scarce, based mainly on small study populations undergoing one type of surgery, with only few of them using the FI as a construct for the preoperative evaluation of this condition [10, 11]. In this regard the current developments with the use of electronical medical records could allow the preoperative estimation of a FI in different settings, supporting the prediction of delirium risk as well as the implementation of tailored delirium prevention management programs. Therefore there is a need to evaluate the FI in this context. Based on data from the control phase before intervention in the PAWEL study (“Patientensicherheit, Wirtschaftlichkeit und Lebensqualität” [transl.: “Patient safety, cost-effectiveness, and quality of life”]) addressing the reduction of delirium risk and postoperative cognitive dysfunction after elective procedures in older adults [15], we aim i) to develop a FI based on the accumulation of deficits to be used in a surgical setting, with information collected at admission, and ii) to analyze the association between this FI and the onset of POD in a middle size population of older adults undergoing cardiac as well as non-cardiac elective surgery.

## Methods

### Study population

Briefly, the PAWEL study was conducted in five medical centers in the southwest of Germany between July 2017 and April 2019 with the aim to evaluate an individualized, multi-professional and multimodal delirium and postoperative cognitive dysfunction (POCD) prevention program. Study setting and design have been described in detail in a previous paper [15]. Patients ≥ 70 years undergoing a major elective surgery with a cut-to-suture time ≥ 60 min were eligible, with the exception of those requiring emergency medicine, with an expected survival ≤ 15 months, or who (or the legal guardian) did not provide written consent. A total of 1470 patients were recruited. For this analysis only data from the control group, defined as PAWEL-R (*n* = 899 with no delirium intervention), was considered to prevent potential bias due to intervention. Due to missing data occurring in 1.8% of the 30 variables considered for the FI, 198 participants had to be excluded (Additional Table 1). In total, 701 participants could be considered for further analysis. The majority of this group was recruited in orthopedic/other surgical wards (*n* = 405, 57.8%), while 296 (42.2%) patients underwent cardiovascular surgery.

Ethical approval for the PAWEL Study was obtained from the Ethics Commission of the Faculty of Medicine of the Eberhard-Karls University and University Hospital Tuebingen (517/2017BO1), Ulm University (425/17) and the Ethics Commission of the University of Potsdam (38/2017). All participants gave written informed consent. PAWEL and PAWEL-R (sub-) study were registered on the German Clinical Trials Register (number DRKS00013311 and DRKS00012797, first regristration November 23^rd^ 2017).

#### Frailty

A 30-item FI based on the accumulation of deficits was constructed based on Searle et al. [16] using preoperatively gathered data (see Table 1). Relevant information for the FI represented following domains: sociodemographic, physical function, sensory perception, comorbidities, self-estimated health/emotions, activities of daily living (ADLs), anthropometrics and laboratory measurements. Considering that the study targeted community-dwelling older adults admitted for an elective surgery, a cut-off ≥ 0.2 was chosen to identify frail subjects [16].

**Table 1:** Variables included in the 30-item Frailty Index

Item	Variable	Coding
**Social situation**		
1	Living alone	0 pt. = no, 1 pt. = yes
**Physical function**		
2	Falls in last 3 months	0 pt. = no, 1 pt. = yes
3	Mobility (MNA)	0 pt. = goes out, 0.5 pt. = able to get out of bed/chair but does not go out, 1 pt. = bed or chair bound
**Sensory tests**		
4	Visual acuity test	0 pt. = correct results, 1 pt. = no correct results
5	Whisper test	0 pt. = both sides correct, 0.5 pt. = one side correct, 1 pt. = none correct
**Comorbidities – case history**		
6	Arterial hypertension	0 pt. = no, 1 pt. = yes
7	Cardiovascular (MI, heart failure, circulatory disorder in feet, arrhythmia, stroke, cerebral hemorrhage, hemiplegic)	0 pt. = no, 1 pt. = yes
8	Neurological (M. Parkinson, seizures)	0 pt. = no, 1 pt. = yes
9	Chronic lung disease	0 pt. = no, 1 pt. = yes
10	Dementia	0 pt. = no, 1 pt. = yes
11	Gastrointestinal ulcer	0 pt. = no, 1 pt. = yes
12	Osteoarthritis	0 pt. = no, 0.5 pt. = hand/other joint, 1 pt. = hand and other joint
13	Urinary incontinence	0 pt. = continent or independent and successful management of urine catheter 0.5 pt. = occasional accident 1 pt. = incontinent (catheterized, unable to manage alone)
14	Tumor	0 pt. = no, 1 pt. = yes
15	Diabetes mellitus	0 pt. = no, 0.5 pt. = without EOD, 1 pt. = EOD
16	Liver disease	0 pt. = no, 0.5 pt. = light, 1 pt. = moderate to severe
**Self-estimated health/emotions**		
17	Subjective memory impairment	0 pt. = no, 1 pt. = yes
18	General health (SF-12)	0 pt. = excellent to good, 0.5 pt. = fair, 1 pt. = poor
19	Moderate activities (SF-12)	0 pt. = not limited at all, 0.5 pt. = limited a little, 1 pt. = limited a lot
20	Climbing several flights of stairs (SF-12)	0 pt. = not limited at all, 0.5 pt. = limited a little, 1 pt. = limited a lot
21	Interference by pain (SF-12)	0 pt. = not at all, 0.25 pt. = a little bit, 0.5 pt. = moderately, 0.75 pt. = quite a bit, 1 pt. = extremely
22	A lot of energy (SF-12)	0 pt. = all/most of the time, 0.5 pt. = a good bit/some of the time, 1 pt. = a little/none of the time
23	Downhearted and blue (SF-12)	0 pt. = none/a little of the time; 0.5 pt. = some of the time; 1 pt. = a good bit/most/all of the time
24	Interference with social contacts (SF-12)	0 pt. = none/a little of the time; 0.5 pt. = some of the time; 1 pt. = most/all of the time
**Measurements**		
25	Weight loss during the last 3 months (MNA)	0 pt. = no, 0.5 pt. = 1–3 kg, 1 pt. = > 3 kg
26	GFR (ml/min//1.73m^2^)	0 pt. = ≥ 60; 0.5 pt. = 30 to < 60; 1 pt. = < 30
27	BMI (kg/m^2^)	0 pt. = 18.5 to < 30; 1 pt. = < 18.5 or ≥ 30
28	Anemia	Men: 1 pt. = < 13 g/dL; Women: 1 pt. = < 12 g/dL
29	Polypharmacy	1 pt. = ≥ 5 medications
30	Barthel Index	0 pt. = ≥ 85; 0.5 pt. = 35–80; 1 pt. = 0–30 points

*pt* Points, *MNA* Mini nutritional assessment, *MI* Myocardial infarction, *EOD* End organ damage, *SF-12* Short form 12, *GFR* Glomerular filtration rate, *BMI* Body mass index

### Variables

Preoperatively assessments were performed with interview-based self-reports by trained assessors containing questions about current living situation, falls, mobility, subjective memory impairment and weight loss. Age, sex, smoking state, alcohol consumption, education and type of surgery were defined as co-variables. Smoking was categorized into non-smoker, ex-smoker and current smoker. An alcohol-score was built based on the self-reported consumption of beer, wine and liquors (options between never = 0 and daily = 5) using the following formula: alcohol score = 0.05 × beer consumption + 0.12 × wine consumption + 0.4 × liquor consumption taking into account the percentage of alcohol of a standard drink [17]. Education was categorized into ≤ 10 or > 10 years of education. Comorbidities were evaluated as follows: diabetes mellitus, arterial hypertension, cardiovascular disease (myocardial infarction, heart failure, circulatory disorders in the lower extremities, arrhythmia, stroke and/or cerebral hemorrhage or hemiplegia), chronic lung disease, dementia, osteoarthritis, urinary incontinence, tumor (lymphoma, leukemia, metastasized solid tumor or any tumor disease), neurological diseases (M. Parkinson and/or seizures) and liver disease. For the emotional and mental state, the seven questions of the SF-12 were considered: General health, moderate activities, climbing several flights of stairs, interference of pain with daily activites, having a lot of energy, feeling downhearted and blue and interference of physical health or emotional problems with social activities [18]. Visual and hearing impairment were evaluated with a visual acuity test and a whisper test, respectively. Body mass index (BMI) was calculated using self-reported weight and height. The preoperative creatinine levels were implemented in the CDK-EPI formula [19] to estimate the glomerular filtration rate (GFR). For hemoglobin World Health Organisation recommended and sex-stratified cut-offs for anemia were used [20]. Polypharmacy was present if five or more medications were taken [21]. ADLs were assessed using the Barthel Index [22]. For secondary analysis timed-up-and-go (TUG) and handgrip strength were considered. TUG reflected participant`s time needed to rise from a chair, walk 3 m and return into a sitting position [23]. Assistive devices (e.g. walking stick) were allowed. Cut-offs for TUG were defined as follows: 0 pt.: < 10 s., 0.25 pt.: ≥ 10 and < 20 s., 0.75 pt.: ≥ 20 and < 30 s., 1 pt.: ≥ 30 s. [23]. Handgrip strength was measured with a JAMAR hand dynamometer testing both hands. The second measurement on the stronger one was considered in the FI. Differentiating by sex a deficit was assumed by < 16 kg in women and < 27 kg in men [24].

### Delirium

The incidence of POD was assessed at bedside by performing a daily Confusion Assessment Method (I-CAM) [25, 26] during the first postoperative 7 days. In addition a chart review at discharge was performed by trained research physicians applying the DSM-5 delirium criteria as reference standard [27, 28]. POD was a combined endpoint allocated by a positive I-CAM on any postoperative day and/or detection of delirium during chart review [15].

### Statistical analysis

For descriptive analyses categorical variables were expressed as numbers and percentage, continuous variables as mean (standard deviation) or median (Q1, Q3). We proceeded to identify the top ten contributors to the FI among those undergoing cardiac and orthopaedic/other surgical procedures respectively. We used logistic regression models to evaluate the association between frailty and the onset of delirium using the FI as a continuous variable as well as a categorical one. Subjects with a FI ≥ 0.2 were defined as frail [29]. **Model 1** adjusted for sex and age; **Model 2** adjusted for sex, age, alcohol consumption, smoking, education and type of surgery. We examined the presence of effect modification by sex, and by type of surgery without significant findings (all *p*-values > 0.2). Odds ratios (OR) with their 95% confidence interval (CI) as well as the calculated c-statistic are reported. A secondary analysis excluding those with a length of stay < 7 days, and missing data on postoperative delirium at 2-months follow-up (*n* = 672) was performed. In a subsample of 517 participants without missing data for functional measurements we built a 32-items FI enriched with the TUG and handgrip strength. In order to evaluate the impact of adding functional measurements on the predictive value of the FI a secondary analysis was performed contrasting the results of the 30-items versus the 32-items FI. Additional Fig. 1 shows the definition of the study samples. Statistical significance was set to a *p*-value < 0.05. All calculations were performed using IBM SPSS software version 26. Graphics were made with R software version 3.6.3 (R Foundation for Statistical Computing, Vienna, Austria; www.R-project.org).

## Results

Baseline characteristics of the study population (*n* = 701) stratified by frailty are shown in Table 2, with a mean age of 77.1 (SD 4.7) years and 52.4% male participants. Overall 296 (42.2%) patients underwent cardiac and vascular surgery, while 405 (57.8%) patients were treated in orthopedic or other surgical departments. A total of 165 (23.5%) POD were diagnosed, with a statistically significant higher prevalence among cardiac patients (108 POD, 36.5%) when compared to orthopedic/other surgical patients (57 POD, 14.1%) (Additional Table 2). Among 165 POD cases only 14 (8.5%) were detected only using the I-CAM, with 93 (56.4%) cases detected through the chart review, and 58 (35.2%) noticeable for delirium in both instruments. On average patients stayed 9 days, with 77 (11.0%) and 11 (1.6%) patients discharged in less than 7 and 3 days, respectively. Compared to cardiac surgery, orthopedic/other surgical patients were older, more often female and more likely to live alone. They also showed less alcohol consumption, a lower functional status (higher rate of falls in the last 3 months, lower mobility, lower Barthel Index), worse hearing ability, a higher prevalence of dementia, cancer and urine incontinence as well as a poorer self-estimated health. However, they showed less polypharmacy, a better GFR and a lower incidence of POD (Additional Table 2).

**Table 2:** Participant’s characteristics stratified by frailty

			Total (*n* = 701)	**Frailty Status**	
				Non-frail (*n* = 173)	Frail (*n* = 528)
**Delirium, n (%)**			165 (23.5)	31 (17.9)	134 (25.4)
**Type of surgery, n (%)**	Cardiac		296 (42.2)	94 (54.3)	202 (38.3)
	Orthopedic		405 (57.8)	79 (45.7)	326 (61.7)
**Age (years), mean (SD)**			77.1 (4.7)	75.6 (4.5)	77.5 (4.7)
**Male, n (%)**			367 (52.4)	106 (61.3)	261 (49.4)
**Education > 10 years, n (%)**			139 (19.8)	47 (27.2)	92 (17.4)
**Alcohol score**^a^**, median (Q1|Q3)**			0.34 (0.12|0.69)	0.41 (0.17|0.86)	0.29 (0.05|0.62)
**Living alone, n (%)**			193 (27.5)	32 (18.5)	161 (30.5)
**Fall in last 3 months, n (%)**			106 (15.1)	9 (5.2)	97 (18.4)
**Mobility (MNA), n (%)**	Goes out		638 (91.0)	172 (99.4)	466 (88.3)
	Gets out of bed/ chair, does not go out		57 (8.1)	1 (0.6)	56 (10.6)
	Bed or chair bound		6 (0.9)	0	6 (1.1)
**Comorbidities, n (%)**	Dementia		11 (1.6)	0	11 (2.1)
	Tumor		162 (23.1)	21 (12.1)	141 (26.7)
	Urinary incontinence	None	633 (90.3)	167 (96.5)	466 (88.3)
		Occasional	60 (8.6)	6 (3.5)	54 (10.2)
		Incontinent	8 (1.1)	0	8 (1.5)
	Hypertension		533 (76.0)	98 (56.6)	435 (82.4)
	CVD		441 (62.9)	72 (41.6)	369 (69.9)
	Chronic lung disease		80 (11.4)	12 (6.9)	68 (12.9)
	GI ulcer		67 (9.6)	6 (3.5)	61 (11.6)
	Osteoarthritis	Mono	281 (40.1)	58 (33.5)	223 (42.2)
		Poly	122 (17.4)	18 (10.4)	104 (19.7)
	Diabetes mellitus	No EOD	139 (19.8)	20 (11.6)	119 (22.5)
		With EOD	27 (3.8)	0	27 (5.1)
	Liver disease	Light	40 (5.7)	5 (2.9)	35 (6.6)
		Moderate to severe	16 (2.3)	2 (1.2)	14 (2.7)
**Sensory deficits, n (%)**	Visual		140 (20.0)	22 (12.7)	118 (22.3)
	Whisper Test	Only one side correct	135 (19.3)	21 (12.1)	114 (21.6)
		None correct	157 (22.4)	28 (16.2)	129 (24.4)
**Weight loss, n (%)**	1 to 3 kg		133 (19.0)	23 (13.3)	110 (20.8)
	> 3 kg		140 (20.0)	10 (5.8)	130 (24.6)
**GFR (ml/min/1.73m**^**2**^**), median (Q1|Q3)**			71.8 (57.2|85.0)	75.9 (65.8|86.2)	70.3 (53.7|83.8)
**BMI (kg/m**^**2**^**), median (Q1|Q3)**			26.8 (24.1|29.9)	25.7 (23.4|28.1)	27.2 (24.4|30.4)
**Anemia**^**b**^**, n (%)**	Female		72 (21.6)	3 (4.5)	69 (25.8)
	Male		114 (31.1)	15 (14.2)	99 (37.9)
**Polypharmacy, n (%)**			467 (66.6)	69 (39.9)	398 (75.4)
**Barthel Index (points)**^c^**, n(%)**	≥ 85		636 (90.7)	173 (100.0)	463 (87.7)
	35 to 80		56 (8.0)	0	56 (10.6)
	0 to 30		9 (1.3)	0	9 (1.7)
**Subjective memory impairment, n (%)**			344 (49.1)	51 (29.5)	293 (55.5)
**General health (SF-12), n (%)**	Excellent to good		453 (64.6)	159 (91.9)	294 (55.7)
	Fair		208 (29.7)	14 (8.1)	194 (36.7)
	Poor		40 (5.7)	0	40 (7.6)
**TUG (sec), median (Q1|Q3) (n = 555)**			12.0 (9.0|16.0)	10.0 (8.0|12.5)	12.0 (9.6|18.0)
**Handgrip strength (kg), median (Q1|Q3)**	Female (*n* = 299)		21.0 (18.0|25.0)	23.0 (18.0|26.0)	21.0 (17.0|24.0)
	Male (*n* = 334)		36.0 (30.0|42.0)	38.0 (32.5|43.5)	34.0 (30.0|41.3)
**Length of stay, median (min, q1, q3, max)**			9 (2,8,12,174)	9 (3,8,11,56)	10 (2,8,13,74)

*MNA* Mini nutritional assessment, *CVD* Cardiovasculary disease, *GI* Gastrointestinal disease, *EOD* Endorgan damage, *GFR* Glomerular filtration rate, *BMI* Body mass index, *Polypharmacy* ≥ 5 long-term medications, *SF-12* Short form 12, *TUG* Time-up-and-go-test

^a^alcohol score = 0.05*(beer amount) + 0.12*(wine amount) + 0.4*(spirits amount)

^b^anemia: male hemoglobin < 13 g/dl; female < 12 g/dl

^c^https://kcgeriatrie.de/Assessments_in_der_Geriatrie/Seiten/Bereich_-_Selbstversorgung.aspx

### Frailty Index

The FI showed a right-skewed distribution (Additional Fig. 2). We observed a median FI of 0.27 [IQR 0.06, 0.65]. Notably 528 of 701 participants (75.3%) were identified as frail (FI ≥ 0.2). Orthopedic/other surgical patients yielded on average higher FI values (median 0.28; Q1 0.21|Q3 0.36) and were more often identified as frail (80.5%) than cardiac surgery patients (median 0.23; Q1 0.18|Q3 0.30; 68.2% frail). Median FI values were higher for women (median 0.28; Q1 0.21| Q3 0.37) than for men (median 0.25; Q1 0.18 | Q3 0.32) with 79.9% of women identified as frail versus only 71.1% of men (Table 3). The following nine deficits were identified among the top ten contributors to the FI for all patients, independently of the type of surgery: arterial hypertension, cardiovascular disease and osteoarthritis as comorbidities; taking ≥ 5 medications; reporting physical limitation by climbing several flight of stairs and/or performing moderate activities; having little levels of energy, subjective memory impairment or decreased hearing (Additional Table 3).

**Table 3:** Descriptive characteristics for the frailty index stratfied by type of surgery

	Study population			Cardiac surgery			Orthopedic/other surgery		
	**Overall**	**Female**	**Male**	**Overall**	**Female**	**Male**	**Overall**	**Female**	**Male**
	*n* = 701	*n* = 334	*n* = 367	*n* = 296	*n* = 93	*n* = 203	*n* = 405	*n* = 241	*n* = 164
**Mean**	0.27	0.29	0.26	0.25	0.27	0.24	0.29	0.30	0.28
**Standard deviation**	0.11	0.11	0.10	0.10	0.10	0.10	0.11	0.11	0.11
**Minimum**	0.06	0.06	0.07	0.06	0.06	0.07	0.06	0.06	0.07
**1. Quartile**	0.20	0.21	0.18	0.18	0.19	0.17	0.21	0.21	0.21
**Median**	0.27	0.28	0.25	0.23	0.26	0.22	0.28	0.30	0.28
**3. Quartile**	0.34	0.37	0.32	0.30	0.33	0.28	0.36	0.38	0.36
**Maximum**	0.65	0.57	0.65	0.55	0.55	0.51	0.65	0.57	0.65
**Frail (FI ≥ 0.2), n (%)**	528 (75.3)	267 (79.9)	261 (71.1)	202 (68.2)	70 (75.3)	132 (65.0)	326 (80.5)	197 (81.7)	129 (78.7)
**Non-frail (FI < 0.2), n (%)**	173 (24.7)	67 (20.1)	106 (28.9)	94(31.8)	23 (24.7)	71 (35.0)	79 (19.5)	44 (18.3)	35 (21.3)

We observed 134 incident delirium events among frail subjects representing 25.4%, while only 31 deliriums could be detected among non-frail (17.9%). Compared to non-frail, frail participants were older, more often female, less educated, showed lower alcohol consumption and were more likely to live alone. Poorer physical functioning within the frail group was noticed with a higher rate of falls, lower levels of mobility, lower Barthel Index and worse results in TUG and handgrip strength (if available). Comparing comorbidities frail participants were diagnosed more often with arterial hypertension, cardiovascular disease, chronic lung disease, dementia, gastrointestinal ulcer, osteoarthritis, urinary incontinence, tumor, diabetes mellitus and liver disease than non-frail ones. Frail participants had lower visual and hearing abilities, lower GFR and higher rate of polypharmacy, anemia and higher BMI. In general, frail participants rated their health lower in the SF-12 than fit participants (Table 2).

### Frailty and postoperative delirium

**Model 1** showed a statistically significant association between frailty and POD. Frail participants had higher odds for POD compared to non-frail (OR 1.66 [95% CI 1.06, 2.59]), where a 0.1 increment of the FI was associated with an OR of 1.32 [95% CI 1.12, 1.57] after adjustment for age and sex. After further adjustment in **Model 2** including education, smoking, alcohol-score and type of surgery frailty showed a higher OR for POD (OR 2.14 [95% 1.33, 3.44], with an OR of 1.57 [95% CI 1.30, 1.90] for a 0.1 increase of the FI. The highest c-statistic (AUC) of 0.722 was seen in **Model 2** with frailty as a continuous variable (Table 4).

**Table 4:** Logistic regression for the association between frailty and POD (*n* = 701)

	**Model 1**^**a**^		**Model 2**^**b**^	
	OR [95% CI]	c-statistic	OR [95% CI]	c-statistic
Frailty (categorical) (FI ≥ 0.2)	1.66 [1.06, 2.59]	0.596	2.14 [1.33, 3.44]	0.705
Frailty Index continuous (increase of 0.1)	1.32 [1.12, 1.57]	0.613	1.57 [1.30, 1.90]	0.722

OR-odds ratio; CI-confidence interval

^a^adjusted for age and sex

^b^adjusted for age, sex, education, smoking, alcohol-score and type of surgery

### Secondary analysis

The observed association between frailty and POD remained when performing the secondary analysis excluding those with a length of stay < 7 days and missing data on postoperative delirium at 2-months follow-up (*n* = 29) with a total of 672 participants without any sign for introduction of bias (Additional Table 4). We were able to build a 32-items FI including functional parameters such as TUG and handgrip strength for 517 of 701 participants. When compared to the study population this subpopulation was slightly younger with a higher ratio of men, cardiac surgery patients, lower rate of polypharmacy and less reported falls (Additional Table 5). No relevant differences in the odds ratios of **Model 2** as well as in the distribution between the 30-items and the 32-items FI could be detected (Table 5, Additional Fig. 3). After adding parameters for muscle strength to the 30-items FI the OR for those frail (FI ≥ 0.2) went from 1.95 [95% CI 1.17, 3.25] to 1.80 [95% CI 1.11, 2.91] after adjustment for age, sex, education, smoking, alcohol consumption and type of surgery. Furthermore, no improvement was observed in model’s discrimination (both c-statistics 0.69). As a continuous variable, a 0.1 increment of the FI including parameters of muscle strength entailed an OR of 1.66 [95% CI 1.31, 2.09] compared to an OR of 1.61 [95% CI 1.28, 2.02] when using the 30-items FI, without any improvement in the discrimination (both c-statistic 0.72).

**Table 5:** Secondary analysis: Logistic regression for the association between frailty and POD within the sub-population (*n* = 517)

	**Model 2**^**a**^			
	Frail versus non-frail		Frailty Index (continuous)	
	OR [95% CI]	c-statistic	OR^b^ [95% CI]	c-statistic
Frailty Index without functionality (30 items)	1.95 [1.17, 3.25]	0.690	1.61 [1.28, 2.02]	0.715
Frailty Index with functionality (32 items)	1.80 [1.11, 2.91]	0.688	1.66 [1.31, 2.09]	0.718

OR-odds ratio; CI-confidence interval

^a^adjusted for age, sex, education, smoking, alcohol score, type of surgery

^b^OR for any 0.1 increment in the frailty index

## Discussion

Our data showed a positive association between frailty defined by a 30-item FI based on the accumulation of deficits and the onset of POD among older adults undergoing elective surgery. Our secondary analysis adding measured parameters for muscle strength (TUG and handgrip strength) to the FI did not show an improvement of model’s discrimination. Hence, a 30-item FI based on variables routinely obtained preoperatively is a significant predictor to identify those at high risk for the onset of POD.

The distribution of the FI in our population of older adults undergoing elective surgery is consistent with observations made in literature, with women having on average higher frailty indices than men [30]. Contrary, the observed prevalence of frailty based on a model of accumulation of deficits using a cut-off of 0.2 is markedly higher than the one reported in the literature as well as the one expected in community-dwelling older adults [10]. In this context available data on prevalence of frailty in patients undergoing elective surgery is mainly based on the phenotypic model of frailty, making a direct comparison impossible. In addition, our study sample already represents a subpopulation of older adults with limited health conditions requiring an elective surgical intervention, which may explain the observed high prevalence of frailty. In our study cardiac surgery patients were less likely to be identified as frail than orthopedic/general surgery patients. However, the 62.5% frailty prevalence among cardiac surgery PAWEL population matches observations made in another study using a similar approach with a 35-item FI in patients undergoing cardiac surgery, where 66.2% participants were classified as frail (FI ≥ 0.2) [31].

With respect to the association with delirium our results are also consistent with the one reported in a recent metaanalysis including 11 studies using different definitions of frailty with an overall accumulative adjusted OR of 2.45 [95% CI 1.58, 3.81] in frail patients undergoing elective surgery [10]. Another metaanalysis including 9 studies representing 794 patients undergoing surgery showed an adjusted OR of 2.14 [95% CI 1.43, 3.19] [11]. Both meta-analyses report challenges in relation to the heterogeneity of the study populations, the type of surgery and the assessments used for the definition of frailty and the identification of delirium [9], so that further studies are needed in order to evaluate the association for different frailty concepts in different settings. The obtained c-statistics of 0.70 and 0.72, when using the FI as a categorical and a continuous variable, respectively, show a moderate discriminatory power in predicting postoperative delirium. These results are of similar magnitude as the discriminatory power reported recently for the Fried Phenotype Model with a c-statistic of 0.73 [32]. When considering only patients undergoing cardiac surgery, markedly higher OR for POD has been reported (OR 3.98 [95% CI 1.10, 14.38] when compared to our results with an OR of 2.00 [95% CI 1.19, 3.40] (data not shown), even in a similar setiting with a high prevalence of frailty according to the model of accumulation of deficits [31], emphasizing the need of further studies.

The detection of POD remains a challenge. Our results showed that overall 72 cases (43.6%) could be identified through the I-CAM alone, highlighting the limitation of this screening instrument for the capture of delirium, a fluctuating condition. The observed prevalence of POD is consistent with the one reported in the literature, with a notably higher prevalence of POD among those undergoing cardiac surgery when compared to other types of surgeries. This difference has been associated to the complex interplay between predisposing factors such as the underlying cardiovascular disease, diabetes, low ejection fraction, elevated creatinine, postoperative atrial fibrillation, pneumonia, extracardiac arteriopathy [33] or environmental cofactors like ICU noise/light as well as the systemic inflammation with endothelial dysfunction and blood–brain barrier disruption reported after the use of cardiopulmonary bypass causing neuroinflammation [34].

POD is a well-known risk factor for further complications of surgery such as extended length of stay or institutionalization [5]. Surgical teams usually get to know their patients at the time of admission, so that an evaluation of frailty based on functional parameters or functional status weeks before surgery, as attempted in the clinical frailty scale, can become a challenge. Taking advantage of the overall information collected at admission to systematically build a FI could help to identify patients with a high-risk for POD, which might benefit from preventive interventions addressing modifiable risk factors and from rescheduling or modifiying treatment approaches [35]. Such interventions may include involvement of geriatricians in perioperative patient care as well as delirium-specialised staff, deprescription of pro-delirious medication (e.g. sedative-hypnotic drugs) [2], or other interventions such as the one implemented in the intervention group participating in the PAWEL study [15, 36].

### Strengths and limitations

Our analysis included a large number of participants (*n* = 701), had a multicenter setting (5 medical centers) and evaluated different surgical types (cardiac and non-cardiac), allowing a more differentiated analysis. Moreover, two approaches helped to assess precisely postoperative delirium prevalence: I-CAM, a modified version of the commonly used CAM, and a Chart Review, which allowed to detect any delirium that might have been missed by I-CAM. Combining those two methods helped to keep the rate of missed delirium with its fluctuating course low. The proposed 30-items FI fulfills the requirement of multidimensionality as proposed by Searle et al. [16]. Because of missing baseline data we were able to build the FI only for 78.0% of the study population. Those excluded were noted to be more often living alone, to have a higher prevalence of falls in the last three months, with less prevalence for hypertension, visual or bilateral auditory impairments, and good functionality according to the Barthel Index (Additional table 6). Nevertheless, and although FI items are considered with the same weight in the model of accumulation of deficits, the observed distribution of the FI, its sex-specific variability along ages as well as the observed estimates for the association with delirium and the discriminatory power of the models, all of them consistent with the available literature, can be seen as indicators for a good construct of the index for this study population.

Unfortunately, only 57.5% of the population had data for functional physical parameters addressing muscle strength, known to be a good predictor for different outcomes and a surrogate for physical frailty in older adults [37]. Therefore we were able to evaluate the predictive value of a 32-item FI including TUG and handgrip strength only in a subsample of 517 patients. This secondary analysis within this subsample showed no further improvement in model’s discrimination after adding them, making the 30-item FI more feasible during preoperative evaluation since it contains mostly routinely gathered data not requiring the performance of extra time-consuming tests. Moreover, it can also be used for bed-bound patients (e.g. for hip surgery or fracture of femoral neck), which is a huge benefit compared to frailty assessments using mobility variables. In addition, and contrary to the clinical frailty scale, which captures a time frame [38], a prior clinical interaction with the patient is not needed in order to build a FI based on patient’s characteristics at the time of admission reinforcing its utility in surgical wards. To which extent our FI would be able to describe a relationship between frailty and delirium in geriatric or internal medicine settings could and should be analysed in further studies.

## Conclusion

According to multiple and international societies the assessment of frailty prior to a surgery has been recommended [32]. Our 30-items FI based mostly on data, defined as a routine in geriatric patients and collected preoperatively, could serve for the identification of patients undergoing elective surgery being at high risk for the onset of postoperative delirium. Further addition of parameters for muscle strength such as TUG and handgrip strength to the FI did not improve discrimination of the model. Available electronical medical records could support the automatic calculation of a FI in surgical wards, helping surgeons to improve the management of patients at high risk for POD.

## Supplementary Information

**Additional file 1.**

## Data Availability

Study data were collected and managed by using SecuTrial® electronic data capture tools hosted at Interactive Systems GmbH and supervised by the University of Potsdam. The datasets used and/or analyzed during the current study are available from the corresponding author on reasonable request.

## Abbreviations

ADL: Activities of Daily Living
CI: Confidence Interval
FI: Frailty Index
GFR: Glomerular Filtration Rate
I-CAM: Confusion Assessment Method
ICU: Intensive Care Unit
OR: Odds ratios
POD: Postoperative Delilrium
TUG: Timed-up-and-go
