# Supplementary material for: Frailty index and its association with the onset of postoperative delirium in older adults undergoing elective surgery
Source: BMC Geriatr. 2023 Feb 11;23:90. doi: 10.1186/s12877-022-03663-7 (PMC9921654; doi:10.1186/s12877-022-03663-7)
Supplement: Supplementary file 1 — Additional file 1. [file 12877_2022_3663_MOESM1_ESM.docx]

**Frailty Index and its association with the onset of postoperative delirium in older adults undergoing elective surgery – the PAWEL Study.**

Janina Steenblock^1,2,3,4^, Ulrike Braisch^1,2,5^, Simone Brefka^1,2,3^, Christine Thomas^6,7,8^, Gerhard W. Eschweiler^9^, Michael Rapp^10^, Brigitte Metz^11^, Christoph Maurer^12^, Christine A. F. von Arnim^13^, Matthias. L. Herrmann^7,9^, Sören Wagner ^8,14^, Michael Denkinger^1,2,3,4^, Dhayana Dallmeier^1,2,4,15^.

**Author affiliations**

^1^Agaplesion Bethesda Clinic, Ulm, Germany

^2^Geriatric Center Ulm/Alb-Donau, Ulm, Germany

^3^Institute for Geriatric Research, University Clinic Ulm, Ulm, Germany

4Medical Faculty, Ulm University, Ulm, Germany

^5^Institute of Epidemiology and Medical Biometry, Ulm University, Ulm, Germany

^6^Department of Old Age Psychiatry and Psychotherapy, Klinikum Stuttgart, Stuttgart.

^7^ Department of Neurology and Neurophysiology, Medical Center-University of Freiburg, Freiburg, Germany.

^8^ Department of Anesthesia, Critical Care and Pain Medicine, Beth Israel Deaconess Medical Center, Harvard Medical School, Boston, MA 02215, USA

^9^Geriatric Center at the University Hospital Tuebingen, Tuebingen, Germany.

^10^Department of Social and Preventive Medicine, University of Potsdam, Am Neuen Palais 10, 14469 Potsdam, Germany.

^11^Geriatric Center Karlsruhe, ViDia Christian Clinics Karlsruhe, Karlsruhe, Germany.

^12^Center for Geriatric Medicine and Gerontology, University of Freiburg, Germany.

^13^Department of Geriatrics, University Medical Center Göttingen, Göttingen, Germany.

^14^Department of Anaesthesiology, Klinikum Stuttgart, Kriegsbergstrasse 60, 70174 Stuttgart.

^15^Department of Epidemiology, Boston University School of Public Health, Boston, USA

**Additional files**

**Additional figure 1: Study population.**

Study population (FI 30 items)

n = 701

PAWEL-R study population

n = 899

n = 198 participants with missing data for variables considered in the 30-items FI

Sub-population for secondary analysis including functional measurements (FI 32 items)

n = 517

n =184 participants with missing data for functional measurements

| **Additional table 1.** Missing data for the frailty index variables, n (%) | | | | |
| --- | --- | --- | --- | --- |
|  |  | **Study sample**  **n=899** | **Cardiac**  **n=337** | **Orthopedic/others**  **n=562** |
| **Item** | **Variable** |  |  |  |
|  | **Social situation** |  |  |  |
| 1 | Living alone | 0 (0.0) | 0 (0.0) | 0 (0.0) |
|  | **Physical function** |  |  |  |
| 2 | Falls in last 3 months | 1 (0.11) | 0 (0.0) | 1 (0.18) |
| 3 | Mobility (MNA) | 20 (2.22) | 2 (0.59) | 18 (3.20) |
|  | **Sensory test** |  |  |  |
| 4 | Visual acuity test | 42 (4.67) | 7 (2.08) | 35 (6.23) |
| 5 | Whisper test | 16 (1.78) | 2 (0.59) | 14 (2.49) |
|  | **Comorbidities – case history** |  |  |  |
| 6 | Arterial hypertension | 0 (0.0) | 0 (0.0) | 0 (0.0) |
| 7 | Cardiovascular (MI, heart failure, circulatory disorder in feet, arrhythmia, stroke, cerebral hemorrhage, hemiplegic) | 0 (0.0) | 0 (0.0) | 0 (0.0) |
| 8 | Neurological (M. Parkinson, seizures) | 0 (0.0) | 0 (0.0) | 0 (0.0) |
| 9 | Chronic lung disease | 0 (0.0) | 0 (0.0) | 0 (0.0) |
| 10 | Dementia | 0 (0.0) | 0 (0.0) | 0 (0.0) |
| 11 | Gastrointestinal ulcer | 0 (0.0) | 0 (0.0) | 0 (0.0) |
| 12 | Osteoarthritis | 0 (0.0) | 0 (0.0) | 0 (0.0) |
| 13 | Urinary incontinence | 19 (2.11) | 3 (0.89) | 16 (2.85) |
| 14 | Tumor | 0 (0.0) | 0 (0.0) | 0 (0.0) |
| 15 | Diabetes mellitus | 0 (0.0) | 0 (0.0) | 0 (0.0) |
| 16 | Liver disease | 0 (0.0) | 0 (0.0) | 0 (0.0) |
|  | **Self-estimated health/emotions** |  |  |  |
| 17 | Subjective memory impairment | 21 (2.33) | 5 (1.48) | 16 (2.85) |
| 18 | General health (SF-12) | 26 (2.89) | 5 (1.48) | 21 (3.74) |
| 19 | Moderate activities (SF-12) | 26 (2.89) | 5 (1.48) | 21 (3.74) |
| 20 | Climbing several flights of stairs (SF-12) | 26 (2.89) | 5 (1.48) | 21 (3.74) |
| 21 | Interference by pain (SF-12) | 28 (3.11) | 5 (1.48) | 23 (4.09) |
| 22 | A lot of energy (SF-12) | 36 (4.00) | 5 (1.48) | 31 (5.52) |
| 23 | Downhearted and blue (SF-12) | 42 (4.67) | 5 (1.48) | 37 (6.58) |
| 24 | Interference with social contacts (SF-12) | 33 (3.67) | 5 (1.48) | 28 (4.98) |
|  | **Measurements** |  |  |  |
| 25 | Weight loss during the last 3 months (MNA) | 62 (6.90) | 7 (2.08) | 55 (9.79) |
| 26 | GFR (ml/min//1.73m^2^) | 19 (2.11) | 3 (0.89) | 16 (2.85) |
| 27 | BMI (kg/m^2^) | 3 (0.33) | 1 (0.30) | 2 (0.36) |
| 28 | Anemia | 17 (1.89) | 4 (1.19) | 13 (2.31) |
| 29 | Polypharmacy | 28 (3.11) | 6 (1.78) | 22 (3.91) |
| 30 | Barthel Index | 22 (2.45) | 3 (0.89) | 19 (3.38) |
|  | Total missing (30*899=26970, cardiac 30*337=10110; 30*562=16860) | 487 (1.8) | 78 (0.77) | 409 (2.42) |

| Additional table 2: Participant’s characteristics differentiated by type of surgery. | | | | | |
| --- | --- | --- | --- | --- | --- |
|  | |  | Total  (n=701) | **Type of surgery** | |
|  |  |  |  | Cardiac  (n=296) | Orthopedic/others  (n=405) |
| Delirium, n (%) | |  | 165 (23.5) | 108 (36.5) | 57 (14.1) |
| Age (years), mean (SD) | |  | 77.1 (4.7) | 76.4 (4.6) | 77.5 (4.8) |
| Male, n (%) | |  | 367 (52.4) | 203 (68.6) | 164 (40.5) |
| Education >10 years, n (%) | |  | 139 (19.8) | 61 (20.6) | 78 (19.3) |
| Alcohol score, median (Q1\|Q3) | |  | 0.34 (0.12\|0.69) | 0.39 (0.12\|0.85) | 0.34 (0.12\|0.60) |
| Living alone, n (%) | |  | 193 (27.5) | 64 (21.6) | 129 (31.9) |
| Fall in last 3 months, n (%) | |  | 106 (15.1) | 29 (9.8) | 77 (19.0) |
| Mobility (MNA) | Goes out |  | 638 (91.0) | 284 (95.9) | 354 (87.4) |
|  | Gets out of bed/ chair, does not get out |  | 57 (8.1) | 11 (3.7) | 46 (11.4) |
|  | Bed or chair bound |  | 6 (0.9) | 1 (0.3) | 5 (1.2) |
| Comorbidities, n (%) | Dementia |  | 11 (1.6) | 0 (0.0) | 11 (2.7) |
|  | Tumor |  | 162 (23.1) | 51 (17.2) | 111 (27.4) |
|  | Urinary incontinence | None | 633 (90.3) | 282 (95.3) | 351 (86.7) |
|  |  | Occasional | 60 (8.6) | 10 (3.4) | 50 (12.3) |
|  |  | Incontinent | 8 (1.1) | 4 (1.4) | 4 (1.0) |
|  | Hypertension |  | 533 (76.0) | 242 (81.8) | 291 (71.9) |
|  | CVD |  | 441 (62.9) | 226 (76.4) | 215 (53.1) |
|  | Chronic lung disease |  | 80 (11.4) | 35 (11.8) | 45 (11.1) |
|  | GI ulcer |  | 67 (9.6) | 25 (8.4) | 42 (10.4) |
|  | Osteoarthritis | Mono | 281 (40.1) | 80 (27.0) | 201 (49.6) |
|  |  | Poly | 122 (17.4) | 36 (12.2) | 86 (21.2) |
|  | Diabetes | No EOD | 139 (19.8) | 63 (21.3) | 76 (18.8) |
|  |  | With EOD | 27 (3.8) | 15 (5.1) | 12 (2.9) |
|  | Liver disease | Light | 40 (5.7) | 13 (4.4) | 27 (6.7) |
|  |  | Moderate to severe | 16 (2.3) | 5 (1.7) | 11 (2.7) |
| Sensory deficits, n (%) | Visual |  | 140 (20.0) | 51 (17.2) | 89 (22.0) |
|  | Whisper Test | Only one side correct | 135 (19.3) | 41 (13.9) | 94 (23.2) |
|  |  | None correct | 157 (22.4) | 62 (20.9) | 95 (23.5) |
| Weight loss, n (%) | 1 to 3 kg |  | 133 (19.0) | 63 (21.3) | 70 (17.3) |
|  | >3kg |  | 140 (20.0) | 56 (18.9) | 84 (20.7) |
| GFR (ml/min/1.73m^2^), median (Q1\|Q3) | |  | 71.8 (57.2\|85.0) | 71.3 (56.8\|83.3) | 72.3 (57.8\|85.8) |
| BMI (kg/m²), median (Q1\|Q3) | |  | 26.8 (24.0\|29.9) | 26.9 (24.1\|29.7) | 26.7 (24.1\|30.0) |
| Anemia _b_, n (%) | Female |  | 72 (21.6) | 25 (26.9) | 47 (19.5) |
|  | Male |  | 114 (31.1) | 60 (29.6) | 54 (32.9) |
| Polypharmacy, n (%) | |  | 467 (66.6) | 213 (72.0) | 254 (62.7) |
| Barthel Index (points), n (%) | ≥85 |  | 636 (90.7) | 288 (97.3) | 348 (85.9) |
|  | 35 to 80 |  | 56 (8.0) | 8 (2.7) | 48 (11.9) |
|  | 0 to 30 |  | 9 (1.3) | 0 (0.0) | 9 (2.2) |
| Subjective memory impairment,  n (%) | |  | 344 (49.1) | 137 (46.3) | 207 (51.1) |
| General health (SF-12) | Excellent to good |  | 453 (64.6) | 221 (74.7) | 232 (57.3) |
|  | Fair |  | 208 (29.7) | 58 (19.6) | 150 (37.0) |
|  | Poor |  | 40 (5.7) | 17 (5.7) | 23 (5.7) |
| TUG (sec), median (Q1\|Q3) (n = 555) | |  | 12.0 (9.0\|16.0) | 10.0 (8.1\|12.7) | 13.0 (10.0\|19.0) |
| Hand grip strength, median (Q1\|Q3) | Female (n = 299) |  | 21.0 (18.0\|25.0) | 22.0 (18.0\|26.0) | 21.0 (17.0\|25.0) |
|  | Male (n = 334) |  | 36.0 (30.0\|42.0) | 36.0 (30.0\|42.0) | 34.3 (28.0\|40.7) |
| Length of stay (days), median (min, q1, q3, max) | |  | 9  (2,8,12,74) | 9  (3,8,12,57) | 10  (2,8,12,74) |
| MNA = Mini nutritional assessment; CVD = cardiovascular disease; GI = gastrointestinal, EOD = end organ damage; Auditory = both sides incorrect; GFR = glomerular filtration rate; BMI = body mass index; Polypharmacy = ≥ 5 long-term medications; SF-12 = Short form 12; TUG = time-up-and-go-test  _a_ alcohol score = 0.05*(beer amount) + 0.12*(wine amount) + 0.4*(spirits amount)  _b_ anemia: male haemoglobin <13 g/dl; female <12g/dl  _c_ [https://kcgeriatrie.de/Assessments_in_der_Geriatrie/Seiten/Bereich_-_Selbstversorgung.aspx](https://deref-web-02.de/mail/client/V52PiE3oWwQ/dereferrer/?redirectUrl=https%3A%2F%2Fkcgeriatrie.de%2FAssessments_in_der_Geriatrie%2FSeiten%2FBereich_-_Selbstversorgung.aspx) | | | | | |

**Additional figure 2:** Distribution of frailty index (N=701).


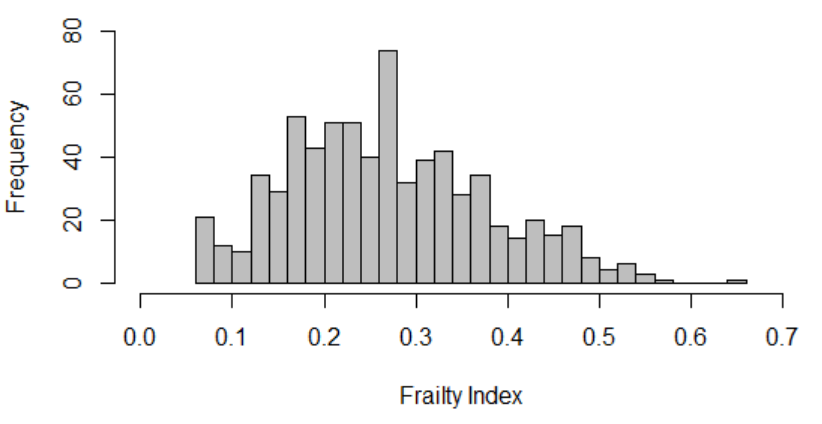


| **Additional table 3:** Contributing factors to the frailty index separated by surgery type and sorted by percentages (i.e. proportion of subjects with $\geq$0.5 points in each variable of the frailty index) in descending order. | | | |
| --- | --- | --- | --- |
| **Type of surgery** | | | |
| **Cardiac** | | **Orthopedic/others** | |
| **%** | **Frailty index variables sorted by frequencies in descending order** | | **%** |
| 81.8 | Arterial hypertension | Climbing several flights of stairs (SF-12) | 80.0 |
| 76.4 | Cardiovascular disease | Moderate activities (SF-12) | 74.1 |
| 72.0 | Polypharmacy | Interference by pain (SF-12) | 72.6 |
| 67.2 | Climbing several flights of stairs (SF-12) | Arterial hypertension | 71.9 |
| 56.4 | A lot of energy (SF-12) | Osteoarthritis | 70.9 |
| 48.3 | Moderate activities (SF-12) | Polypharmacy | 62.7 |
| 46.3 | Subjective memory impairment | A lot of energy (SF-12) | 60.5 |
| 40.2 | Weight loss during the last 3 months (MNA) | Cardiovascular disease | 53.1 |
| 39.2 | Osteoarthritis | Subjective memory impairment | 51.1 |
| 34.8 | Whisper test | Whisper test | 46.7 |
| 32.4 | Interference by pain (SF-12) | General health (SF-12) | 42.7 |
| 30.4 | GFR (ml/min/1.73m2) | Interference with social contacts (SF-12) | 38.5 |
| 28.7 | Anemia | Downhearted and blue (SF-12) | 38.3 |
| 27.7 | Downhearted and blue (SF-12) | Weight loss during the last 3 months (MNA) | 38.0 |
| 26.4 | Diabetes mellitus | Living alone | 31.9 |
| 25.3 | General health (SF-12) | Tumor | 27.4 |
| 24.0 | BMI (kg/m2) | GFR (ml/min/1.73m2) | 27.2 |
| 21.6 | Living alone | BMI (kg/m2) | 26.2 |
| 17.9 | Interference with social contacts (SF-12) | Anemia | 24.9 |
| 17.2 | Visual acuity test | Visual acuity test | 22.0 |
| 17.2 | Tumor | Diabetes mellitus | 21.7 |
| 11.8 | Chronic lung disease | Falls in last 3 months | 19.0 |
| 9.8 | Falls in last 3 months | Barthel Index | 14.1 |
| 8.5 | Gastrointestinal ulcer | Urinary incontinence | 13.3 |
| 6.1 | Liver disease | Mobility (MNA) | 12.6 |
| 4.7 | Urinary incontinence | Chronic lung disease | 11.1 |
| 4.1 | Mobility (MNA) | Gastrointestinal ulcer | 10.4 |
| 4.1 | Neurological disease (Parkinson) | Liver disease | 9.4 |
| 2.7 | Barthel Index | Dementia | 2.7 |
| 0.0 | Dementia | Neurological disease (Parkinson) | 2.5 |

| **Additional table 4.** Logistic regression for the association between frailty and POD after exclusion of those with a length of stay < 7 days and missing information on POD at 2-months follow-up (n=672). | | | | | | | | |
| --- | --- | --- | --- | --- | --- | --- | --- | --- |
|  | | incident delirium/n (%) | | Model 1_a_ | | | Model 2_b_ | |
|  | |  |  | OR [95% CI] | | c-statistic | OR [95% CI] | c-statistic |
| Frailty (categorical) (FI ≥0.2) | frail: 134/510 (26.3%) | | 1.64 [1.05, 2.58] | | 0.600 | | 2.16 [1.34, 3.49] | 0.712 |
| Frailty Index continuous (increase of 0.1) | 165/672 (24.6%) | | 1.32 [1.11, 1.56] | | 0.615 | | 1.60 [1.32, 1.94] | 0.729 |
| OR-odds ratio; CI-confidence interval  _a_ adjusted for age and sex  _b_ adjusted for age, sex, education, smoking, alcohol-score and type of surgery | | | | | | | | |

| **Additional Table 5:** Participants’s characteristics of the study population (n=701) vs. sub-population (n=517). | | |
| --- | --- | --- |
|  | Study population  (n=701) | Sub-sample for secondary analysis  (n=517) |
| Age (years), mean (SD) | 77.1 (4.7) | 76.6 (4.6) |
| Male, n (%) | 367 (52.4) | 285 (55.1) |
| Cardiac surgery, n (%) | 296 (42.2) | 258 (49.9) |
| Orthopedic surgery, n (%) | 405 (57.8) | 259 (50.1) |
| Polypharmacy*, n (%) | 467 (66.6) | 328 (63.4) |
| Fall in the last 3 months, n (%) | 106 (15.1) | 55 (10.6) |
| Delirium, n (%) | 165 (23.5) | 125 (24.2) |
| *Polypharmacy: ≥ 5 long-term medications | | |

**Additional figure 3:** Distribution of frailty index comparing 30- and 32-items frailty index in sub-population (n=517).


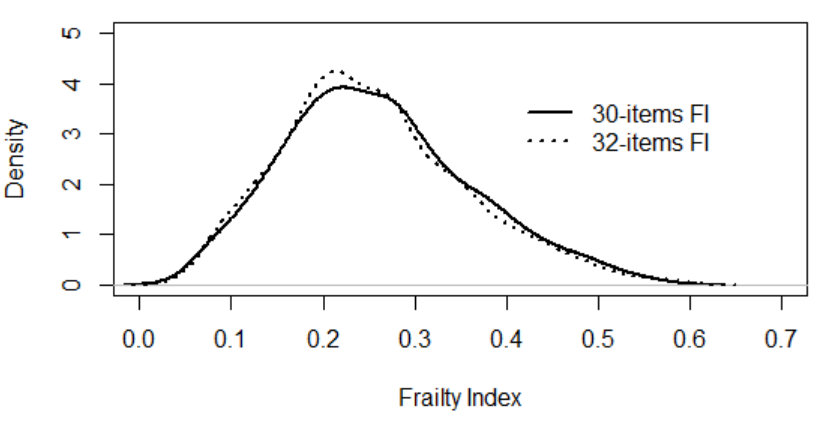


| Additional table 6: Characteristics of study population and excluded population. | | | | | | |
| --- | --- | --- | --- | --- | --- | --- |
|  | |  | | Study population  (n=701) | Excluded population | |
|  | |  | |  | (n=198) | Missings (n) |
| Delirium, n (%) | |  | | 165 (23.5) | 44 (22.2) | 0 |
| Age (years), mean (SD) | |  | | 77.1 (4.7) | 78.2 (5.2) | 0 |
| Male, n (%) | |  | | 367 (52.4) | 88 (44.4) | 0 |
| Education >10 years, n (%) | |  | | 139 (19.8) | 32 (16.3) | 2 |
| Alcohol score, median (Q1\|Q3) | |  | | 0.34 (0.12\|0.69) | 0.36 (0.12\|0.62) | 7 |
| Living alone, n (%) | |  | | 193 (27.5) | 71 (35.9) | 0 |
| Fall in last 3 months, n (%) | |  | | 106 (15.1) | 47 (23.9) | 1 |
| Mobility (MNA), n (%) | Goes out | |  | 638 (91.0) | 152 (85.4) | 20 |
|  | Gets out of bed/ chair, does not go out | |  | 57 (8.1) | 20 (11.2) |  |
|  | Bed or chair bound | |  | 6 (0.9) | 6 (3.4) |  |
| Comorbidities, n (%) | Dementia | |  | 11 (1.6) | 3 (1.5) | 0 |
|  | Tumor | |  | 162 (23.1) | 42 (21.2) | 0 |
|  | Urinary incontinence | | None | 633 (90.3) | 155 (86.6) | 19 |
|  |  | | Occasional | 60 (8.6) | 19 (10.6) |  |
|  |  | | Incontinent | 8 (1.1) | 5 (2.8) |  |
|  | Hypertension | |  | 533 (76.0) | 135 (68.2) | 0 |
|  | CVD | |  | 441 (62.9) | 111 (56.1) | 0 |
|  | Chronic lung disease | |  | 80 (11.4) | 26 (13.1) | 0 |
|  | GI ulcer | |  | 67 (9.6) | 16 (8.1) | 0 |
|  | Osteoarthritis | | Mono | 281 (40.1) | 84 (42.4) | 0 |
|  |  |  | Poly | 122 (17.4) | 30 (15.2) |  |
|  | Diabetes | | No EOD | 139 (19.8) | 31 (15.7) | 0 |
|  |  |  | With EOD | 27 (3.8) | 9 (4.5) |  |
|  | Liver disease | | Light | 40 (5.7) | 6 (3.0) | 0 |
|  |  |  | Moderate to severe | 16 (2.3) | 1 (0.5) |  |
| Sensory deficits, n (%) | Visual | |  | 140 (20.0) | 45 (28.8) | 42 |
|  | Whisper Test | | Only one side correct | 135 (19.3) | 51 (28.0) | 16 |
|  |  | | None correct | 157 (22.4) | 43 (23.6) |  |
| Weight loss, n (%) | 1 to 3 kg | |  | 133 (19) | 21 (15.4) | 62 |
|  | >3kg | |  | 140 (20.0) | 26 (19.1) |  |
| GFR (ml/min//1.73m^2^), median (Q1\|Q3) | |  | | 71.8 (57.2\|85.0) | 69.9 (55.2\|82.7) | 19 |
| BMI (kg/m²), median (Q1\|Q3) | |  | | 26.8 (24.1\|29.9) | 26.1 (23.9\|29.7) | 3 |
| Anemia _b_, n (%) | Female | |  | 72 (21.6) | 28 (27.5) | 8 |
|  | Male | |  | 114 (31.1) | 16 (20.3) | 9 |
| Polypharmacy, n (%) | |  | | 467 (66.6) | 111 (65.3) | 28 |
| Barthel Index (points), n (%) | ≥85 | |  | 636 (90.7) | 143 (81.3) | 22 |
|  | 35 to 80 | |  | 56 (8.0) | 23 (13.1) |  |
|  | 0 to 30 | |  | 9 (1.3) | 10 (5.7) |  |
| Subjective memory impairment, n (%) | |  | | 344 (49.1) | 88 (49.7) | 21 |
| General health (SF-12) | Excellent to good | |  | 453 (64.6) | 94 (54.7) | 26 |
|  | Fair | |  | 208 (29.7) | 65 (37.8) |  |
|  | Poor | |  | 40 (5.7) | 13 (7.6) |  |
| TUG (sec), median (Q1\|Q3) (n = 555) | |  | | 12.0 (9.0\|16.0) | 12.6 (8.9\|17.7) | 73 |
| Hand grip strength, median (Q1\|Q3) | Female (n = 299) | |  | 21.0 (18.0\|25.0) | 20.0 (17.0\|26.0) | 33 |
|  | Male (n = 334) | |  | 36.0 (30.0\|42.0) | 36.0 (28.3\|45.0) | 20 |
| MNA = Mini nutritional assessment; CVD = cardiovascular disease; GI = gastrointestinal, EOD = end organ damage; Auditory = both sides incorrect; GFR = glomerular filtration rate; BMI = body mass index; Polypharmacy = ≥ 5 long-term medications; SF-12 = Short form 12; TUG = time-up-and-go-test  _a_ alcohol score = 0.05*(beer amount) + 0.12*(wine amount) + 0.4*(spirits amount)  _b_ anemia: male haemoglobin <13 g/dl; female <12g/dl  _c_ [https://kcgeriatrie.de/Assessments_in_der_Geriatrie/Seiten/Bereich_-_Selbstversorgung.aspx](https://deref-web-02.de/mail/client/V52PiE3oWwQ/dereferrer/?redirectUrl=https%3A%2F%2Fkcgeriatrie.de%2FAssessments_in_der_Geriatrie%2FSeiten%2FBereich_-_Selbstversorgung.aspx) | | | | | | |
